# Environment, Tropical Disease, and Scientific Networks in Argentina: *Folclore* and Multiscalar Mobilities

**DOI:** 10.1007/s10739-025-09830-x

**Published:** 2025-10-03

**Authors:** Lily Balloffet

**Affiliations:** University of California, Santa Cruz, USA

**Keywords:** Field practice, Tropical medicine, Networks, Mobilities, Latin America

## Abstract

This article employs space and place as analytic categories in the history of life sciences and public health research in rural Argentina. The historical-ecological panorama of South America’s “Gran Chaco” region served as the backdrop to key life sciences research agendas and public health initiatives of the twentieth century. Through an examination of the cross-disciplinary works of Argentine zoologist, schoolteacher, and fiction writer Jorge Washington Ábalos (1915-1979), this investigation reveals how his social and professional identities, together with multiscalar mobilities and networks of knowledge production, came to bear on the ways in which he conducted research, engaged with collaborators during his field practice, and produced knowledge over the course of his career. More broadly, this study posits the sociospatial and cultural specificities of place (in this case the Gran Chaco) as key forces that shape ideas and practices relevant to the history of biology. The study draws from a diverse bibliography of publications from Ábalos’ multifaceted career. His works include scientific reports on the venomous fauna of the Gran Chaco (animal behavior, morphology, and taxonomy), and epidemiological studies of what the global health community today terms “Neglected Tropical Diseases.” These are accompanied by a collection of novels, short stories and autobiographically-inspired accounts of his time spent living in rural landscapes and social contexts.

*Vaulted by quebracho trees, the trail was a maw that enveloped my youth. The forest assumed the condition of a monster; a monster with the persistence of centuries, of all time. That earlier self changed in bearing, and it was no longer I who had ventured into the forest, but rather the forest who had swallowed an intruder.* (Ábalos 1975, p. 11)^1^

## Introduction: Space, Place, and the Field Sciences in Rural Argentina

This article utilizes space and place as analytic categories to explore historical encounters between humans and animals in the Gran Chaco region of Argentina, and the ways in which these dynamics formed an important context for life sciences and public health research. A study of one biologist’s early experiences amidst the ecologies and toxicities of animals such as arachnids, disease-bearing insects, and venomous reptiles reveals layers of moving people, animals, objects, and expertise. These bodies and ideas in motion constituted a historical-ecological panorama which served as the backdrop to key research agendas and initiatives that were a central preoccupation of 20th century life sciences and public health communities in the Gran Chaco region, and far beyond its borders. Through an examination of the cross-disciplinary works of Argentine zoologist, schoolteacher, and fiction writer Jorge Washington Ábalos (1915–1979), a story emerges that highlights the way in which his social and professional identities came to bear on the ways in which he conducted research, engaged with collaborators during his field practice, and produced knowledge over the course of his career. The following contrapuntal analysis of Ábalos’ literary works and scientific research practice illuminates a network of individuals involved in the production of knowledge about human-animal conflict, natural history field methods, and their relationship to public health in the region. Although the Gran Chaco is the specific focal point for this study, this is also a study of movement and interconnection between one of Argentina’s most rural areas with a broader geography of biological and epidemiological research related to the history of Tropical Medicine. It is a story of professional trajectories marked deeply by place and interpersonal experiences, and the ways in which they were tied to evolving infrastructures of public health and education in the 20th century.

The diverse bibliography of publications that resulted from Ábalos’ multifaceted career serves as the backbone of archival materials analyzed for this study. His works include scientific reports on the venomous fauna of the Gran Chaco (animal behavior, morphology, and taxonomy), and epidemiological studies of what the global health community today terms neglected tropical diseases. These are accompanied by a collection of novels, short stories and autobiographically-inspired accounts of his time spent living in rural landscapes and social contexts. Such a notably varied bibliographic terrain invites a multi-pronged approach to this history of life science field practice—one that illuminates how the particularities of place anchored a young scientist’s understanding of his research from his career’s inception onward. Rooting the study in Argentina’s Gran Chaco region also invites not only a decentering of Global North geographies and actors, but also an emphasis on Argentina’s rural spaces in the production of scientific knowledge. This rendering stands in marked contrast with a historic emphasis on metropolitan academic epicenters of research and intellectual life.

Since the 19th century, generations of Argentine intellectuals cast rural spaces as foils to the progress and modernity embodied by Argentina’s cities—particularly the hegemonic Federal Capital of Buenos Aires. Nowhere is this tendency encapsulated more simply than the common, enduring characterization of rural, provincial Argentina as the “*interior*.” This rendering of the *capital vs. interior* is historically laden with presumptions of backwardness, and it preoccupied generations of intellectuals and architects of national political projects dating back to Argentina’s independence from Spanish colonial rule in the early 19th century. Noting the rhetorical broadness of the term *interior*, cultural critics, scholars, and travel writers have aptly referred to perceptions of the Gran Chaco region as being *el interior del interior* (the interior of the interior)—the most rural of rural spaces that Argentina has to offer. By the conclusion of this article, a contrapuntal reading of Ábalos’ literary and scientific works will render a geography of human-nonhuman entanglements in the Gran Chaco as a model for seeing this rural Argentine space as a nexus of connection and mobility. This historical perspective on the Gran Chaco is one of integration in key milestones in the history of life sciences research and public health initiatives in Argentina (and beyond), and highlights the *interior del interior* as an important geography of biological research and knowledge production in the twentieth century.

This study of a young teacher and budding scientist at work in Argentina’s so-called *interior del interior* foregrounds the manifold ways in which the sociospatial specificity of a place—its unique landscape of flora, fauna, and human denizens—shapes people, ideas, and practices relevant to the history of biology. The particularities of time and place shaped Ábalos’ trajectory since his early days as a school teacher, but this story from the Chaco also came to be as a result of transnational networks of actors and institutions. This simultaneous force exerted by micro/local and macro/global conditions and mobilities is a key framework for a broader field of historians of science and technology who have posited national actors and institutions as nodes in transnational networks of “aspiration, expertise, and affiliation of various kinds” that determine “itineraries of knowledge” (Krige 2019, p. 8). Over the past several decades there has been growing scholarly interest in the production and circulation of knowledge at a variety of scales, including the forms of mobilities and knowledge production related to fieldwork. Recent work in the history of field practice looks at these histories as venues for studying intertwined local and transnational perspectives on knowledge production, and seeks to situate the places where life sciences fieldwork is carried out within networks of transportation and communication (Vetter 2011, p. 6).

These kinds of mobilities are central to the story of Ábalos and the various creatures that he studied. Highlighting the generative nature of these mobilities is in keeping with trends in the broader field of the history of science, wherein by the turn of the 20th–21st century, historians saw the sciences as a global phenomenon shaped by means of transnational circulation of knowledge and material culture, as well as through collaborative networks and actors, both social and institutional (Barahona and Raj 2022). In this regard, this article contributes to scholarship that takes the circulation of knowledge as its central analytical hinge point. Increasingly, this historiography has come to emphasize the social nature of knowledge, and incorporate histories of education systems and cultures as vectors by which knowledge travels from person to person and across borders and ultimately over great distances. Ábalos provides the perfect case study, in this sense, in that he was a teacher first and foremost, who then transformed into a scientist. In short, Ábalos himself was deeply concerned with the transmission and translation of knowledges as they related to public health and the life sciences.

In centering the multispecies entanglements between people and venomous or disease-vector animals in the Gran Chaco, this article responds to calls for better integrating histories of science with environmental history. Recent efforts to bring these fields together have taken a dialectic approach to science, technology, and environment that has allowed scholars to pose questions about relations between human and non-human actors, and in the process elucidate the “more-than-human” contexts in which individuals and communities produce knowledge. In doing so, the possibility arises for new narratives to emerge that go beyond traditional frameworks that reiterate the “degradation, declension, and exploitation” of Latin American environments (Chastain and Lorek 2020, p. 6; see also Carey 2009). These efforts to bring beyond-human subjects to the fore complement other decentering impulses which are shaping these and other fields related to histories of science and public health. Namely, historians are looking to engage in the larger project of dismantling simplistic core-versus-periphery frames for charting the directional flows of knowledge and expertise that made possible the production and circulation of scientific knowledge and expertise. To do this, historians are decentering the state and state-centric periodization, emphasizing multiple knowledges, and focusing instead on illuminating “polycentric networks” and “creative interplay” between centers and peripheries—in Latin American contexts, and beyond (Chastain and Lorek 2020, p. 303–330; see also Cueto and Palmer 2015). Whereas two decades ago James Secord called for historians of science to conceptualize science as a form of communication, scholars today have critically responded by stressing the production of scientific practice, knowledge, instruments and techniques as not merely communication or passive circulation, but products that derive from processes of encounter, power and resistance, negotiation, and reconfiguration (Secord 2004; Raj 2013). These efforts to decenter histories of science, and problematize the notion of “international science” in a way that gets away from the center/periphery binary reveal how entanglements between the local and transnational render legible how scientific practices, ideas, materials, and scientists themselves “circulate beyond geographic and epistemic borders” (Barahona and Raj 2022, p. 3). Rather than scientific knowledge and practice diffusing from center to periphery in a classic hub and spokes model, these scholars emphasize how face-to-face contact (especially in the context of field sites) leads to the transformation of researchers and systems of knowledge co-production. Beyond histories of the field sciences, these critical approaches reflect recent scholarship across a range of historical subfields related to the story of Ábalos and his movements through the Gran Chaco, including histories of medicine and public health in Latin America. Collectively, this scholarship seeks to move past a center-periphery model that is itself a byproduct of Cold War developmentalist paradigms, and in so doing adopt a nuanced vision of the ways that people, things, and ideas in motion shape environments and scientific communities. It is in this vein that we may come to understand how a schoolteacher’s engagement in local oral and material cultures of the Gran Chaco—in sum, elements of the region’s *folclore*—molded his career in the life sciences.

## Point of Departure: El Gran Chaco

The so-called Gran Chaco is a subtropical ecoregion that stretches from eastern Bolivia, western Paraguay, and Southwestern Brazil into Argentina. Two major rivers—the Pilcomayo and the Bermejo—flow southeastward through this transborder territory, and help to delineate subregions. North of the Pilcomayo are the plains (*llanos*), dry thorny forests, and palm stands of the *Chaco Boreal*. Between the two waterways are the transitional wetland and woodland ecosystems of the *Chaco Central*. South of the Bermejo, the climate gets wetter, and the landscape gives way to the alluvial lowland plains of the *Chaco Austral*. Collectively the ecoregion hosts an extraordinary diversity of flora and fauna, and represents a critical reservoir of biodiversity for South America. Together with the sheer vastness of the physical space that constitutes the Gran Chaco, these factors have attracted a long history of extractive industrial enterprises. For all its diverse flora and fauna, the Gran Chaco is not wilderness, but something closer to what environmental historians term a “working landscape,” characterized by being highly rural but peopled, with a mixed land use pattern of rural agriculture and small settlements. Historian of science Robert Kohler notes that in the context of working landscapes “the rules and customs of science change […] which is one reason why the field sciences are so interesting,” for “in the sciences of working landscapes, humans are not to be excluded as externalities but rather as objects of investigation along with other natural things” (Kohler 2011, p. 223–224). As a working landscape, the Gran Chaco requires us to treat nature, ecology, and human culture as part of a unified framework. Intentionally situating this study in the Gran Chaco region aligns with a growing move for historians of science to take up regional level objects of study.^2^ When it comes to the Gran Chaco, scholars have long seen the region as one of simultaneous processes of fomentation and marginalization. The Gran Chaco is part of Argentina’s broader Northwestern region—an area that geographer Eric Carter aptly characterizes as a place constructed by metropolitan Argentine elites as a “pathologized geographic imaginary,” in which they viewed endemic poverty, disease, and social ills as mutually reinforcing and difficult to disentangle (Carter 2012, p. 6). At the same time, it was also a region whose network of institutions related to public health benefited Argentine state building efforts in the 19th century, and fostered fruitful networks of scientific sociability over the course of the 20th century (see Bragoni and Míguez 2010; Dimas 2022; Podgorny et al. 2024; Cicerchia et al. 2015).

Starting in the mid-19th century, timber, cotton, and cattle-ranching industries brought an increase in incursions into the Indigenous communities of the Gran Chaco. Prior to that moment, some three dozen tribal groups that populated the region had long resisted the religious and labor systems that were the bedrock of colonial Spanish rule. Thus, the density of settler colonial populations (and the industrial enterprise that accompanied them) in the Gran Chaco remained extremely sparse until the dual-pronged advent of Argentina’s boom in railroad infrastructure and international immigration of the mid-19th century. It was at that point that an onslaught of transformative agricultural, industrial, and labor practices began to unfold in the Gran Chaco—one that would mark the livelihood of its human and beyond-human inhabitants. As it turned out, the tannin-rich wood of abundant *quebracho* trees (genus *Schinopsis*) made for excellent railroad ties and fencing stakes. Logging and rail crews churned through the timber of *quebracho* forests, converting the flora of the natural landscape into the physical infrastructure of rail transport and large-scale ranching. This had a severe impact on the access of Indigenous peoples to their territories, and by the dawn of the 20th century the trifecta of low-cost state lands, access to an increasingly impoverished labor force, and a lack of environmental controls created the ideal conditions for the ongoing extraction of resources that characterizes the region to the present day. Today, the Gran Chaco is experiencing the highest rates of lowland deforestation on the continent (see Hirsch et al. 2021). This developing scenario of environmental degradation and territorial dispossession constituted a critically important context for Ábalos when he arrived there as a young school teacher in 1934.

“What type of place is the Gran Chaco?” mused Argentine anthropologist Gastón Gordillo in a recent volume on the identities, politics and environment of the region. He went on to clarify: “to be more precise, what places constitute, in their diversity, this vast and transnational spatial entity in the tropical lowlands of South America?” (Gordillo 2021, p. 277). Any attempt to apprehend an adequate answer to this question must be grounded in an examination of what he would go on to refer to as the “constitutive rupture” that defined the Gran Chaco—the process by which the region went from being a holdout of Indigenous autonomy to a sacrifice zone of extractive industry in three nation-states (Argentina, Paraguay, and Bolivia). In considering the spatiality of the Chaco, Gordillo recalled decades of ethnographic work, and described being struck by “the distinct social and historical texture” of the stories shared with him by his Indigenous Qomle’ec (Toba) hosts:

I was particularly captivated by the memories of elders who described how back in the 1910s and 1920s that same terrain on the Pilcomayo River was a radically different place under the control of their ancestors, whom they described as proud, egalitarian people who moved freely over a wide area and did not recognize the Argentine state […] Those were memories, in short, about what today seems unthinkable: a territory ‘outside’ of the state. (Gordillo 2021, p. 278)

The palpably recent (and violent) imposition of state power in the Gran Chaco was the “constitutive spatial and territorial feature” of the place that greeted Ábalos upon his arrival to the province of Santiago del Estero in the Chaco Austral (Gordillo 2021, p. 278). There, he would begin his career as a rural school teacher at the helm of a series of one-room schoolhouses. For the next eight years (1934–1942), he worked in a succession of rural, Quechua-speaking communities up and down the Dulce and Salado Rivers. During this time, he became drawn into the social and ecological settings inhabited by his young students. Meanwhile, his own encounters with the particular flora and fauna of the Gran Chaco impelled him toward the kinds of inquiry and practice that would later define his scientific career.

## On the Banks of the Dulce and Salado

The headwaters of the Dulce and Salado Rivers are found in the Andean peaks of Argentina’s Salta Province. From there, each waterway winds southward to the Chaco Austral. The Dulce River’s name (sweet river) comes from the local *Santiagueño* Quechua name, *Mishqui Mayu* (*mishki* sweet, *mayu* river). Cutting diagonally across the province, it ends in the great saline drainage basin of Córdoba province known as *Mar Chiquita* (little ocean) lagoon. The so-called flood pulse of the Dulce—its annual cycle of inundation and drought-controls a lateral exchange of water, nutrients, and organisms between the river’s channel and its surrounding floodplain. Rather than periodic catastrophes, the Dulce’s regular floods represent the river’s most biologically productive feature. The flood pulse moves oxygen and nutrients in and out of the floodplain ecosystem, creating hospitable conditions for hibernation, spawning, and waystations along migration routes.

Like the Dulce, the Salado gains its name from the local Quechua “Cachimayo”(*cachi,* salt, *mayu* river). Unlike the Dulce, which cuts southward from the Andes until it eventually dumps into a giant saltwater lagoon, upon arriving in the Chaco Austral the Salado branches into several arms that fan out across a shallow riverbed. The flood pulse of the Salado creates broad areas of marshland across a swath of the Chaco Austral, but the communities where Ábalos worked lay across the area of the Salado’s most impoverished flow (UNESCO 2007). The families who sent their children to his classrooms in the communities of Tacañitas, Pampa Llastac, Puente Negro, La Costa, and Doña Lorenza dealt with long dry periods—whole years even—during which people plants and animals withered and thirsted.^3^ Compounding that regular scarcity was a 2-year long drought from 1935 to 1937, barely a year in to the young schoolteacher’s tenure in the public education apparatus of rural Santiago del Estero.

Bookending the literary career of Ábalos are two novels that, in a series of vignettes, grapple lyrically and reflectively with his years as a rural school teacher. *Shunko* and *Shalacos*, published in 1949 and 1975, were to be the first two installations in a trilogy of narrative works, but he passed away before completing *Coshmi*, which would have been the final work of the series. In each, the subject, aesthetic, and linguistic interplay between Spanish and Quechua were rooted in *chaqueño* social and ecological worlds and folkloric orality. Though he wrote *Shalacos* as a sequel to *Shunko*, the temporality of the second novel is not a linear continuation on from the first. Rather, each work departs from the same point of inception: the arrival of a young *maestro rural* (rural school teacher) at his first post—a one-room schoolhouse in the Chaco.

From the first to the second novel, time doubles back to this point of departure/arrival, unfolding once more through the narrator’s portraits of schoolchildren and their teacher. These nested stories recount the travails of existence in the ruggedly unforgiving climate and terrain of the Chaco, while simultaneously depicting the landscape as teeming with life (human and otherwise). In *Shalacos*, Ábalos depicts the much-anticipated arrival of the river at the end of a long dry season:

‘Let’s go see the water, *señor*!’ say the children, excited […] In truth, what attention span can I expect for reading, doing arithmetic, or listening to stories about Christopher Columbus, or some region of the globe called ‘Africa,’ when water, that fiercely precious commodity, is right here, at our very door? (Ábalos 1975, p. 69)

The *maestro*—a narrativized autobiographical rendering of Ábalos himself—concedes and lets the students lead him along the dry riverbed, in the direction of the advancing swell of water.

We walk alongside the water. The unevenness of the riverbed finds its level. When the current comes to a low place, it fills it, overflows, and continues onward. Ahead of the swell tumble forth a multitude of spiders, crickets, and other insects—even scorpions, all evicted from their dwellings by the surge. Many of them reach the shore, others are swept away. (Ábalos 1975, p. 70)

This is one of countless scenes from Ábalos’ writings that foreground the role of the natural environment in marking the rhythm of the years that he spent with his pupils in the Chaco. The particular ecology of the place contoured both his experience, and his pedagogy. It also set the stage for the panorama of public health issues faced by the communities in which he worked—issues that later would become subjects of inquiry in Ábalos’ career in the life sciences.

The *maestro* in *Shunko* and *Shalacos* kept a close watch for any signs of illness or acute failure to thrive among his pupils. He treated cases of Trachoma with zinc and copper sulfate eye drops, helped to run off rabid dogs with his shotgun, and administered “injections to stave off *peste*” (possibly yellow fever vaccines) (Ábalos: 1959, p. 49; 1975, p. 84). “Trachoma is a more pressing reality than literacy or arithmetic” mused the *maestro*, and yet “the doctor doesn’t come here, as he is stretched too thin to cover the extent of the zone assigned to him” (Ábalos 1975, p. 73). At the time, these kinds of administrations were part of the regular slate of duties for rural Argentine school teachers who helped fill in the gaps in an insufficient health infrastructure. Through the 19th century and up until the 1950s, Argentine doctors were unable to meet medical demands or offer a broad range of treatments, and teachers participated in efforts to prevent and treat disease (Dimas 2022, p. 8; Curto et al. 2013, p. 102). In 1938, the Argentine National Congress passed Law 12.588 which stipulated the allocation of resources to train rural teachers in the diagnosis and treatment of common ailments such as trachoma. The National Education Council, together with the Justice Ministry and National Hygiene Department coordinated to provide training to rural *maestros* (Congreso de la Nación 1938).

Soon after taking up his first teaching posts, Ábalos began to submit articles and manuscripts to publishers. The genres of his writing ranged from fiction to natural history, and formal academic reports on various aspects of rural life. Collectively, the body of his publications point to a growing conviction that he held regarding the interconnectedness of education and public health. In 1937, *El Monitor de la Educación Común* published an article by Ábalos that shed light on some of his early practices as a school teacher in the Chaco. The *Monitor* is Argentina’s oldest education journal (est. 1881), and was intended to improve the circulation of information and ideas between public schools across the country. The journal was founded at a moment of reform and expansion of the politics of public education in Argentina, and aimed to serve as a news organ that would convey everything from policies to the latest in education-related research alongside a dose of edifying cultural content. It was the official publication of the Ministry of Education, and its pages were a mix of translated texts by internationally-known pedagogues such as Maria Montessori, writings by Latin American literati, opinion pieces, field reports from classroom teachers, and research reviews. When Ábalos’ article appeared in the *Monitor*, he was serving as the Director of School No.502 in the community of Tacañitas, isolated on the parched floodplain of the Salado River. Keeping up with the *Monitor*’s potpourri of Education-related content may well have served him as a way of staying abreast of a broader public sphere of pedagogical and cultural conversations.

The article that he published there was not, however, a treatise on teaching methods or philosophy. Rather, his contribution titled “Venomous Snakes of Santiago del Estero” was an overview of venomous snakebite as a public health challenge in rural communities like Tacañitas. In the text, he walked the reader through an overview of basic snake morphology and key behavioral traits of the most common venomous snakes that a rural school teacher would be likely to encounter. Though his personal experiences and practices were not the focus of the article, which aimed at a more general informational tone, glimpses nevertheless appeared of Ábalos’ day to day interactions with the local venomous fauna. When asserting that the rattlesnakes (*Crotalus durissus*) were the most ill-tempered of the venomous snakes in the Chaco, he revealed that he had taken to collecting live specimens which he maintained in small cages in his one-room home. “They quickly become irritated, which makes them all the more dangerous,” he wrote, noting that his own specimens “showed their irritation by rattling their tail at the mere sound of [him] rolling over in bed” (Ábalos 1937, p. 55). In his description of coral snakes (*Micrurus corallinus*) he noted the regional Quechua name for this brilliantly colored red, white, and black banded serpent, *Tanta Micha.* His inclusion of the local name aligned with Ábalos’ broader commitment to learning the Quechua language in order to be able to effectively communicate with his students, their families and community.

In *Shalacos*, one vignette portrays the moment when several students taught their *maestro* how to identify the *Tanta Micha*, and differentiate it from a similarly colored but harmless species (1975, p. 100). This illustrated the dual relationship that Ábalos articulated in the narrative voice of *Shalacos*, wherein the *maestro* reflects the following sentiment:

More than 4 years have passed since my arrival in this place. This pastoral way of living that seemed naive, flat and gray to me at the beginning, has come to life such that it configures a whole new universe [...] I want to remember some of the many things that these children showed me and taught me about their marvelous world. For me, a subtle dividing line has emerged between two roles: in the classroom, I was the one who taught, during recess I was the one who learned. (Ábalos 1975, p. 29)

Nearly forty years after the publication of his *Monitor* article, Ábalos openly acknowledged that his novel, *Shalacos*, was drawn from his own recollections across several schools and communities in the Chaco Austral. The temporality of that novel’s narrative coincides with the moment in Ábalos’ career when he was presiding over the schoolhouse in Tacañitas—when he had been working as a teacher for a handful of years, and was getting to know the lay of the land and its inhabitants.

Collectively, the novels *Shunko,* and *Shalacos*, together with the *Monitor* article indicate that soon after Ábalos began his tenure in Argentina’s rural public schools, he had homed in on the notion that to best serve his pedagogical ends, he needed to immerse himself in the project of learning about the particular natural and social ecology of the *chaqueño* landscape. This approach required a great deal of time dedicated to classroom duties, and many solitary hours of specimen collecting and writing in his natural history journal. Throughout, however, Ábalos was always looking outward, striving to connect what he learned in the Chaco with intellectual and academic circles of knowledge production. In return, members of Argentina’s lettered elite, and leaders in scientific and public health circles began to take notice of the young *maestro* in the Chaco. Santiago del Estero’s most prominent literary and cultural figure at that time, Bernardo Canal Feijoó, applauded this critical engagement with place that characterized Ábalos’ work—as a teacher, writer, and budding naturalist. Five years on from the young *maestro*’s publishing debut in the *Monitor*, Feijoó would write the Foreword to Ábalos’ first novel-length narrative—a first-person autobiographical account titled *Cuentos con y sin víboras* (“Stories with and without Snakes”):

Ábalos is the teacher who discovers the *folclore* of the place where he is posted, and uses it methodically for his functional purposes. I hope that this formulation that I make of his thought is not misunderstood: *folclore* is not—cannot be—an end for him. Rather, in his hands it is a means, an instrument at the service of a pedagogical consciousness with a clearly historical and current vocation. (Ábalos 1949, p. 9)

With this observation of the role of *folclore* in the Ábalos’ writings, Feijoó referred to the prevalence of the particularly *chaqueño* cultural expressions that would go on to characterize his writing over the course of his career.

Vivid in Ábalos prose are the folkloric rhythms and styles of provincial culture rooted in Indigenous cultures and a colonial past that brought together Hispanic, African, and local actors in hierarchical systems of extraction and unique cultural production ranging from dance to music, textile art, poetry and fashion. In Ábalos’ work, these traces came in various forms. Most obvious was the abundant Quechua vocabulary sprinkled throughout his texts and translated in appended glossaries. The use of *coplas*—colloquial couplets replete with clever turns of phrase—was also central to many atmospheric scenes of rural lifeways, and in later years would even make their way into his scientific writings. The *maestro* would regularly exchange *coplas* with his students—a practice that built rapport, and one in which Ábalos clearly took great pleasure (Ábalos 1975, p. 52). Finally, rich description of the flora and fauna native to the Chaco Austral was the bedrock of his texts, both literary, as well as in works that aimed to be informative and scientific, beginning with his 1937 *Monitor* article. In those intervening years between the *Monitor*’s publication of his inventory of venomous snakes, and Ábalos’ arrival on the Argentine literary scene with his first book, events in the *maestro*’s life would launch him on a new career path in the life sciences.

## From Añatuya to Jujuy

One morning in 1938, Ábalos rose painfully from his cot in the rustic cottage where he lived adjacent to the schoolhouse. He noted with gratitude his ability to see sunlight stream through the window, and hear a racket of birds outside. When he went to bed the previous night, he was in rough shape; he had become suddenly and gravely ill following a scorpion sting. Upon surviving the night, he went about his business, attended to his duties. A few days later and more or less reconstituted after his envenomation, he saddled his horse, Doradillo, and made the 40km ride to the nearest medical clinic in the county seat of Añatuya.^4^ Once he arrived, Ábalos checked himself in and announced that he had been stung by a scorpion earlier that week. He had experienced a plethora of side effects, he explained, and wanted to know (for future reference) whether the clinic had access to antivenom serum, or could at least provide him with any informational literature on the area’s venomous scorpions. He presumed that their answer would be “no,” but he had come to the clinic anyway. Just as he suspected, the medical staff had no antivenom, but what they offered him was nevertheless life-changing.

The clinic staff put him in contact with Dr. Salvador Mazza, a prominent Argentine physician and epidemiologist who had an interest in animal venoms and their medical significance. Eager for the contact, Ábalos accepted the offer, and struck up a correspondence with Mazza right away. He was, after all, primed to engage with experts in epidemiology and public health such as Mazza. In his autodidactic journey to learn about the natural history of his surrounding landscape (in particular its venomous fauna, and corresponding implications in public health), he sought out reference materials to try to help him identify the species in his midst. In his *Monitor* article the year before, he had actually mis-identified the Latin name for the local rattlesnakes or “*cascabeles,*”mistakenly referring to them as *Crotalus Horridus* [sic.]—the North American Timber Rattlesnake, rather than its South American counterpart (*C. durissus*). He similarly mistook the Latin name of the local coral snake as *Micrurus Elaps* [sic.]—supplying readers with two genus names rather than the correct binomial nomenclature (*Micrurus corallinus*). His mistakes indicate that while he was yet a novice naturalist, he was actively seeking out literature to help him learn about local species. In addition to consuming reports from national public health research institutes such as the Instituto Bacteriológico in Buenos Aires, he may well have had access to the writings of contemporaries in El Chaco who practiced medicine and wrote about local landscapes.^5^ In his *Monitor* article he cited their protocols for safe management of venomous snakes in captivity, including instructions for how to “milk” them for their venom. In sum, by the time of his scorpion sting, Ábalos was already dedicated to the project of amassing all of the expertise that he could when it came to the epidemiology of human-animal conflicts in the Chaco.

The day that Ábalos appeared at the regional clinic in Añatuya and acquired Mazza’s contact information was an important inflection point in his life. That correspondence hooked the schoolteacher into a community of scientists, doctors, and so-called hygienists (public health workers) that stretched across Northwestern Argentina, and spanned national and continental borders. At the time, Mazza was living in Argentina’s arid northwesternmost province of Jujuy. Born in Buenos Aires, and educated in prestigious institutions there as well as in Europe, Mazza was an elite figure in the field of Argentine medicine. After completing his medical degree, he was sent to Europe to study communicable disease prevention practices by the German and Austrian armies during World War I. From there he went to work under the prominent French bacteriologist Charles Nicolle at the Pasteur Institute in Tunis. Upon his return to South America, he turned his energies toward the issue of the medically neglected Northwestern region. He even managed to convince the National Health Department to sponsor a consultation visit from Nicolle regarding the possibility of establishing a Pasteur-style institute in Jujuy (Leonard 1992, p. 260). In connecting with Mazza, Ábalos was engaging with an active network of people and forms of expertise whose mobilities linked arid Andean plains to bustling laboratories in Paris, and hospital wards in Tunisia, among a plethora of other sites.

When the extremely eager, twenty-three year old Ábalos wrote to Mazza requesting a copy of a study that he had conducted on scorpion envenomation, the young *maestro* may have been surprised by the speed and enthusiasm of the response that he received from the senior physician. Mazza sent a copy of the paper, and an invitation: “Perhaps you could be a collaborator” wrote Mazza, eager to establish a correspondence with a willing co-conspirator in the Chaco region.^6^ Unbeknownst to Ábalos, he had written to Mazza at a time when the hyper-mobile scientist had recently had to confront is own corporeal limitations: he had been diagnosed with a cardiac condition the year prior, and while still relentless in his travels, years of immense and unceasingly vigorous work and travel seem to have been catching up with him (Leonard 1992, p. 267). Perhaps Mazza’s nightly practice of answering written correspondence became that much more important to him as health concerns threatened his indefatigably mobile lifestyle. At any rate, it’s clear from the archives of Mazza’s personal correspondence that Ábalos was by no means the only *maestro* to have contacted him. A 1941 letter from a correspondent in Estancia Garruchos spoke of a “box with some kind of [insect]” found by a student, who had turned in the specimen to their teacher, who had in turn relayed the specimen to Mazza for identification (Sánchez et al. 2010, p. 184). By the late 1930s, Mazza’s leadership was the driving force behind regional public health efforts and research programs. Both required a steady supply of samples collected in the field and relayed to laboratories, from blood draws to soil samples and animal specimens such as the bug from Garruchos.

Starting in 1926, Mazza spearheaded a bacteriological research campaign, the Mission for the Study of Regional Pathologies, or MEPRA (*Misión de Estudios de Patología Regional Argentina).* MEPRA’s charge was to conduct a “study of the diseases that afflict people and animals in the north of the Republic” by identifying the regional presence of diseases, differentiating their clinical presentations, and assessing their prevalence, severity, and priority (Kapelusz-Poppi 2015, p. 509). Following the French colonial Pasteur model, scientists stationed in research facilities across the Global South (e.g. Tunis, Bandung, Dakar) studied the varied, regional nature of pathologies. Following this logic, Mazza and his colleagues at the University of Buenos Aires Medical School established the MEPRA headquarters in the rural provincial capital of San Salvador de Jujuy, inaugurating the new laboratories, medical offices, and library in 1929. Mazza and his supporters saw this as a humanistic and patriotic effort to bring modern science to the *interior*, and the expansion of medical access was a pillar of their campaign. Conveniently, notes historian Ana María Kapelusz-Poppi, “treating local people would also give MEPRA access to the dwellers’ homes as well as to biological samples and epidemiological data.” And finally, following the advice of a colleague who focused on the epidemiology of Malaria, Mazza was fully convinced of the importance of exploring the regional landscape of any given pathology, rather than simply importing a public health plan from another context (Kapelusz-Poppi 2015, p. 499). Though MEPRA lost its government backing following Argentina’s fascist coup of 1930, barely a year after the Jujuy campus opened its doors, Mazza donated personal funds and orchestrated private donations to keep the operation running.

When Ábalos’ missive arrived from the Chaco, Mazza immediately clocked the young *maestro* as a highly promising collaborator. This was a time in which Argentina was experiencing a transition from “hygienism” toward “sanitarism” as the guiding framework in the medical establishment’s approach to public health. Focus was shifting away from the “hygiene” issues of urban and suburban populations (e.g. tuberculosis, cholera, and social ills that came along with tenement housing) (Sánchez 2007). A “sanitation” framework foregrounded a preoccupation with rural populations’ contact with animals and vermin, and identified rural dwellings—*ranchos*—as key sites of encounter between humans, vermin, and disease vector animals (see Sánchez 2007). Conveniently for Mazza, Ábalos was stationed in the Chaco Austral (a subregion relevant to the epidemiologies that interested Mazza, and the sanitarist movement more generally). Furthermore, because of the nature of his work as a schoolteacher—a post which Ábalos elected to fulfill through regular and sustained outreach and engagement with local families—he already had a firmly integrated (or at the very least widely respected and accepted) place in the social fabric of his local community. Crucially, this meant that he had reliable access to the homes of his students and their families. House calls to check on an unwell or otherwise jeopardized student were commonplace for Ábalos, and access to living structures meant the potential for gathering epidemiological data to relay to MEPRA. The regularity of house-calls made to check on, or even directly attend to, the wellbeing of a student is depicted throughout Ábalos’ fictional (yet biographically based) and nonfictional accounts of his years working in *chaqueño* schools. He may not have written as much to Mazza in that first exchange of letters, but the *maestro* was keenly aware of the presence of “regional pathologies” as evidenced by his efforts to inoculate students, treat them for diseases like Trachoma, and even administer “injections” to children suffering the effects of envenomation from snakes (Ábalos 1975, p. 49).

Finally, the fact that the young teacher was unflinching in the face of venomous fauna (indeed, a budding naturalist!) boded well for pressing him into service as a collector and observer of all manner of specimens. Mazza’s own eagerness shone through in the long list of specimens that he rattled off in the letter accompanying the scorpion literature requested by Ábalos: “Perhaps you could collaborate […] by sending armadillos, possums, and ferrets from that region, as well as live snakes and mites if possible, and *vinchuca* bugs from the caves of armadillos and bird nests.”^7^ The final specimens—the *vinchuca* bugs—would provide critical epidemiological data points on a disease that Mazza and his colleagues had been working extremely hard to track and pathologically profile in northern Argentina: American Trypanosomiasis, or Chagas Disease. This request wasn’t outlandish; rather, Argentine schoolteachers were known as collaborators in both public health efforts, as well as instrumental in the building of natural history collections for both provincial and metropolitan museum collections.^8^

For years, Mazza and colleagues at MEPRA and its affiliate organization SAPRN (*Sociedad de Patología Regional del Norte,* Society of Regional Pathologies of the North) had been trying to prove the extent of Chagas Disease’s presence in Argentina. The condition, a parasitosis first identified in 1909 by Brazilian physician Carlos Chagas, is transmitted by blood-sucking insects known locally as “*barbeiros*.” Commonly known in Argentina as *vinchucas,* these insects are the main vectors of this protozoan parasite-borne illness that causes complex and potentially long-term health problems. Ábalos knew all too well that *vinchucas* thrive in the thatched roofs, adobe walls, and unsealed dirt floors typical of poor rural communities across the Gran Chaco. Mazza’s letter couldn’t have been any sharper of an inflection point for the *maestro*. Responding to this entreaty to support the efforts of the MEPRA/SAPRN Chagas Disease study was to become his calling. Forthwith, Ábalos assembled his field kit, and set out into the countryside, the “*monte,”* to collect specimens. This collecting regimen would shape the approaches and strategies that would later inform his field practice in the realms of both entomology and public health, and bring him into contact with new mentors and projects in the life sciences.

## In the Monte

Following Mazza’s directive, Ábalos set to work at once, roving the *monte* and the *ranchos* up and down the Dulce and Salado rivers. He collected animal and insect specimens, and obtained blood samples to relay to the MEPRA labs in Jujuy. A vivid description of the mobile research setup that Ábalos included in his 1942 *Cuentos con y sin víboras* attests to the breadth of collecting and sampling activities that he undertook:

I mounted my bicycle, which was equipped with a carbine lashed to a long pipe, a jar for vipers, my *caspi machajuero*, a backpack full of test tubes, boxes, tweezers, and a small fanny pack of provisions. I dressed in my comfortable *bombachas de campesino*, *alpargatas*, a shirt and a helmet, carrying in my belt a hunting knife, a hatchet, and a string of bottles and jars. I went about the area intent on leaving no nest unprodded, no tree trunk with its bark unexamined, no cave unexplored. I checked ranches for parasites, and when it was possible I drew blood from every child, dog, or cat that I could manage to sample [for Chagas Disease]. For each sample I prepared a microscope slide with a fat drop of blood. (Ábalos 1949, pp. 11–12)

Soon, people began to take notice of his collecting sojourns into the *ranchos* and *monte*, and locals began referring to him as the *maestro “bichero*” (bug collector). “Ah, the *bichero* must have been here” they would say, noting a disturbed spiderweb or rooted out den (Ábalos 1949, p. 12). Ábalos’ description of his kitted-out bicycle and chosen attire paints him as a strange figure, laden down with all manner of implements. And yet, small but intentional details in his description belie that he also saw his field supplies and practices as distinctly *of* the Chaco. He chose the Quechua term *caspi machajuero* to describe the long forked stick that he would use to subdue venomous snakes, and he noted his choice to don typical attire of Argentina’s rural “*interior:*” *bombachas de campesino* (billowing pants gathered at the ankle), and *alpargatas* (peasant shoes of simple fabric). Just as his students had taught him to differentiate a venomous coral snake (*Tanta Micha*) from its harmless counterpart, so too it appears that he learned the local method for employing his *caspi machajuero* to pin a writhing viper before wrestling it into a collection jar. Put another way, it appears that the *chaqueño* “vernacular knowledge” had begun to “trickle up” into the practices of a future entomology expert (see Kohler 2011, p. 216). This entanglement of expert and vernacular knowledges, of human and animal relations—in a sense, a *folclore*-inscribed understanding of his subjects and their social-historical-ecological realms—would go on to be the hallmark of Ábalos’ career.^9^

As he toiled away, running School no. 502 in Tacañitas and moonlighting as the *maestro bichero,* Ábalos stayed in regular communication with Mazza. The professional relationship that grew from that correspondence opened doors for the young school teacher, who at that point still had no formal scientific training, but was eager to be in dialogue with experts in everything from bacteriology to zoology. In this way, he befriended a zoologist with whom he began to correspond about his travails as a teacher-naturalist in the Chaco. He wrote to him about (among other topics) the presence of a velvety black spider that had made its web in the corner of his room. Over time, Ábalos developed a certain fondness for the spider—carefully observing her daily habits, and even going so far as to catch flies for her, gently depositing them on the strands of her web. By then his modest dwelling was crammed with “every kind of creature—in drawers, bottles, jars.” Across the room from the spider corner was his collection of live snakes. On his desk was a profusion of spiders and centipedes “scattered in a pleasing disarray” of vessels (Ábalos 1949, p. 66).

In letters to his zoologist friend, he described the tenderness that he felt toward the little spider, his companion throughout days and nights of remote solitude (“*soledad campesina*”) (Ábalos 1971, p. 21). Concerned, his colleague wrote to him, cautioning the novice naturalist that his housemate was none other than the infamous *arañita de la muerte* (spider of death), a black widow spider of the genus *Latrodectus* whose venom had no antidote*.* Decades later in one of his short stories, Ábalos would reminisce about this moment of realization upon receiving the zoologist’s warning: “How could that beautiful creature be poisonous? How was it possible that its tiny perfect beauty contained a poison that could maim or kill?”(Ábalos 1971, p.22) Stunned and unsettled, he impulsively grabbed a kerosene blowtorch, and on the spot incinerated the arachnid, her web, and an egg sac teeming with young. If the literary scene conveyed finality, however, the reality was anything but. Nearly four decades later, upon his 1975 inauguration into the Argentine National Academy of Sciences, Ábalos closed his speech with the following line: “It’s hard to believe, but I am telling you—that beautiful spider in the corner of my room modified my destiny, and put me definitively on a different path” (Ábalos 1976, p.102).

After the incineration incident, Ábalos began to notice the alarming number of *Latrodectus* spiders in the Chaco. Once aware of the lack of antivenom for their bites, he pressed his zoologist colleague to connect him with someone who might be interested in attempting to create a remedy. The general approach to synthesizing antivenom sera was well established by then, as Ábalos was well aware. He had, in fact, included a detailed rundown on the manufacture and administration of antivenom for treating snakebite in his *Monitor* article but two years prior. He had even tried his hand at “milking” vipers for their venom, the first step in the process by which antivenom sera are made to this day (Ábalos 1937). At Ábalos’ insistence, the zoologist sent him the address for Dr. Bernardo Houssay, a professor of Physiology at the nation’s most prestigious medical school in the Federal Capital, the Universidad de Buenos Aires. In April of 1940, Houssay wrote back to the young schoolteacher who had contacted him out of the blue. He was resoundingly enthusiastic.

Although it had been more than a decade since Houssay had dedicated any of his own research to the topic of venoms and antivenoms, he wrote back immediately to propose a collaboration. It was clearly a topic of interest for him: between 1916 and 1923 he had published thirty-nine articles, reports and informational resources on snake and spider venoms. He wrote about their properties, and summarized assessments of the current landscape and future possibilities for antivenom production and distribution infrastructure nationally. Teachers, he noted in a 1916 report to the National Department of Hygiene, had proven excellent collaborators in the collection of venomous snakes in Brazil, enabling the production of antivenoms at the continent’s leading biologic research center, the Instituto Butantan in São Paulo (Houssay 1916). Houssay put his doctoral student, Rafael Sampayo, in charge of the laboratory work, and informed Ábalos of the raw materials that he hoped the *maestro* could collect for the purpose of synthesizing *Latrodectus* antivenom. “To study the action of the poison [*sic*] and prepare the [antivenom] serum, we will need to be able to count on a large supply of spiders.” Ábalos agreed, promising to send “thousands” of specimens (Huerga 1981, pp. 47–48).^10^ With that, the *maestro bichero* set to work. He already had his collecting setup at the ready after two years of specimen retrieval toward Mazza’s research into the regional pathology of Chagas Disease.

As Ábalos’ *Latrodectus* collection campaign came prior to his formal scientific training, he did not publish reports of his field practices or results in the kinds of journals and bulletins where Houssay sent his flurry of venom studies. He did, however, write vivid accounts of his time spent hunting *arañitas de la muerte.* These first appeared in print in 1942 in the autobiographical *Cuentos con y sin Víboras,* and he repeatedly revisited the *Latrodectus* campaign in the literary writings that he published later in his career. In his first rendition, he described a day of collecting spiders along the Salado River, moving along a transect between the towns of Añatuya and Colonia Dora.

I needed to come up with a thousand poison glands [from *Latrodectus mactans*] for the preparation of an immunizing serum, which was carried out in a lab in Buenos Aires. I was planning to submit my supply of the raw materials that Sunday. I collected specimens by leaning over the L-shaped railing [of a bridge known for its large population of these spiders], and inserting the test tubes into the L’s corner. This would force the *Latrodectus* to enter the tube, and if that didn’t work I carefully plucked them with my tweezers and inserted them manually. This is a task that requires great attention—the trickle of liquid web that *Latrodectus* releases when caught can adhere to the tweezers and create a dangerous situation. Even a small involuntary movement can cause the spider to fall on itself, which can lead to the unpleasant (and serious) consequences of a bite. (1949, pp. 17–18)

Some twenty years later in his 1964 *Norte Pencoso* story collection, he recalled collecting *Latrodectus* at that same bridge site, but in that recounting he expanded his description to include his own practice of venom extraction from his daily haul of specimens.

I remember with nostalgia my ‘ranch-laboratory.’ Spiders captured during the day had to be subjected to venom extraction at night. Serious business. A 500-wick kerosene lamp illuminated the table which I had covered with a large white paper. I expressed the glands by taking the spider out of its tube with one tweezer, and holding it with a second fine tweezer. I then grabbed its fangs and pulled gently to express the venom. The lack of a magnifying glass and the tiny fangs made the task difficult. (Huerga 1981, p.50)

He recalled learning to control his own body to minimize the risk of a bite during this seemingly impossibly minute task (particularly given the lack of any magnification):

It was necessary to control my instinctive movements. I learned to raise my arms gently and withdraw carefully. If I wasn’t deliberate, my actions could make the little spider retreat towards me, pulled by its thread [...] When I think of that era, when the [antivenom] serum had not yet been made, a chill runs down my spine. (Huerga 1981, p. 50)^11^

Risky as it was, the *maestro bichero* managed to nail down a technique that worked, and sent a staggering supply of thousands upon thousands of specimens to Sampayo and Houssay, packed carefully into a glass test-tubes, with each specimen separated from the next by a wad of cotton. This simple barrier, instructed Houssay, would prevent the spiders from eating one another en route from the Chaco to the lab at the Instituto Bacteriológico. Within only a couple of years they were able to successfully synthesize so-called anti-*Latrodectus* serum. These descriptions attest to various themes of recent interest to historians of science, including the sociability practices that connect provincial collectors (including amateurs like Ábalos) with brokers, and go-betweens, and the assembly of laboratory spaces in non-institutional settings such as the *maestro*’s humble hut (see Podgorny et al. 2024; Podgorny and Richard 2023).^12^

Ábalos’ final narrative return to the *Latrodectus* project came in 1978, only a year before his death, when he published an atmospheric but diffuse collection of short stories set against the familiar *chaqueño* backdrop. *La Viuda Negra* (The Black Widow) revealed crucial information regarding the seemingly insurmountable task of collecting the approximately 40,000 spiders over the course of 2 years that were necessary to synthesize an ongoing supply of antivenom. The reality was that it was a collective effort—not merely the zeal of one (albeit tireless) individual. An entire community’s crucial participation in the project comes out in the pages of *La Viuda Negra:*

Our needs for Latrodectus […] are always pressing due to the high demand from the [Bacteriologic Institute] where they inject venom into horses in the serum production process. [My collaborators and I] determined the existence of dense breeding grounds of the spider in various areas [of the Chaco Austral]. From there, local collectors turned out to be effective collaborators. The capture of live females became a small industry that helped the family economy in these regions of temporary exodus where men must go out to the seasonal harvests of neighboring provinces. Those who dedicated themselves to the dangerous task of capturing spiders were mostly women. (Ábalos, 1978, p.11)

Just as valuable as the delicate venom milking techniques that Ábalos practiced in his “ranch-laboratory,” was the experience that he gained in organizing and managing a team of local collaborators in the field. Doubtless his position as the local *maestro* and Director of the Tacañitas schoolhouse aided him immensely in forging this network of contacts. The insight that he gained via his students into the Chaco’s regional rhythms of seasonal migration, and the gendered norms of household management that corresponded with those labor migration patterns, would also have been advantageous as he looked to quickly mobilize the *Latrodectus* collection campaign.

Nearly four decades on, vivid details of that experience stuck with the by-then seasoned entomologist, who at the end of his career served on the faculty of the highly regarded Universidad Nacional de Córdoba. “The Aramayo family were excellent suppliers,” he reminisced. “[The matriarch of the Aramayos] brought the largest spiders, collected in the best conditions, and the most abundant. Our *Latrodectus* venom increased once they started working” (1978, p.12). Supported by families like the Aramayos, the *Latrodectus* campaign success would end up propelling Ábalos out of the Chaco and into the next phase of his career.

## Out of the Chaco—and Many Returns

The synthesis of the nation’s first antivenom for one of its most abundant venomous animals was a noteworthy event. Houssay seized the opportunity to publish a full-page account of the efforts led by the young *maestro* in the science news section of one of the most widely circulated national newspapers, *La Nación.* It was here that, perhaps for the first time, he wasn’t portrayed as a school teacher; rather, Houssay lauded the efforts of the “*joven científico*” (young scientist) (Houssay 1942, p. 50). As a direct result of this news coverage, members of the provincial government of Santiago del Estero took notice, and offered Ábalos a scholarship to relocate to Brazil so that he could study at the region’s most prestigious institution of public health research: the Instituto Oswaldo Cruz in Rio de Janeiro. To cite the illustrious list of individuals who spent time studying and conducting research at the Instituto Oswaldo Cruz would be a tour de force of the region’s most acclaimed scientific minds of the late-19th century onward. Ábalos’ participation in the *Latrodectus* campaign catapulted him into the institutional epicenter for the research and theorization of public health, laboratory practice, and experimental medicine. He would spend a year in Brazil on scholarship, before returning to Argentina to take up the post of entomologist at the Instituto de Medicina Regional (IMR) at the Universidad Nacional de Tucumán (UNT). Though he was offered the title as a result of the universally legible prestige of having spent a year studying at the preeminent Instituto Oswaldo Cruz, the reality is that he had spent years consolidating a network of correspondents, collaborators, and field assistants such as Mazza, Houssay, and the spider collectors of the Gran Chaco. Thus, he effectively laid the groundwork for the field practice that would allow him to execute his official duties as UNT’s entomologist.

As a *maestro* he had built the cultural and linguistic fluency and social networks that would be necessary for him to carry forward a slate of zoological and public health research projects for decades to come. It is only through reading his scientific and literary works in counterpoint, however, that one can glean the depth of this scientist’s commitment to seeing and addressing public health problems in their particular cultural, linguistic, and educational contexts. It is only through his literary writings, for example, that we learn of his fluency in Quechua—a monumentally useful asset for engaging with local subjects in an array of social relations key to his field practice, but invisible in his scientific papers. While architects of the MEPRA worked extensively in multiethnic and multilingual communities and emphasized the regional identity of the institution, Ábalos’ ability to speak Quechua, trade in *coplas*, and productively leverage his understanding of vernacular, folkloric knowledges, went above and beyond those impulses.

Ábalos’ first academic posting in Santiago del Estero’s neighboring province of Tucumán signified a return to many of the familiar textures of provincial Argentine life after his time in the bustling metropolis of Rio de Janeiro, but it was also a major departure. In accepting the post at IMR, he left his career as a *maestro rural,* never to return to the helm of a one-room schoolhouse in the Chaco. His professional departure left him wracked with guilt for having left behind his students and their families—an anguish that he discussed openly in interviews and in his writings for the rest of his life. But this career shift did not translate to physical estrangement from the *llanos* and rivers of the *monte chaqueño.* His career was one of constant return to the Chaco, starting with an intensive schedule of fieldwork to study *vinchucas* and Chagas Disease. His director, Dr. Cecilio Romaña, was a former collaborator with Mazza in his quest to prove the presence and extent of Trypanosomiasis in Argentina, and had himself benefitted from time abroad in the halls of the Parisian Pasteur Institute where he studied tropical medicine. Like Ábalos, he also spent a year at the Instituto Oswaldo Cruz, and may well have crossed paths with the newly arrived *maestro bichero* before returning to Argentina to assume the directorship of the IMR (Sierra e Iglesias 1999). These overlapping circuits attest to the interplay between the specific local geographies of tropical disease pathologies, and the broadly international networks of people and institutions concerned with their study.^13^

**Fig. 1:**
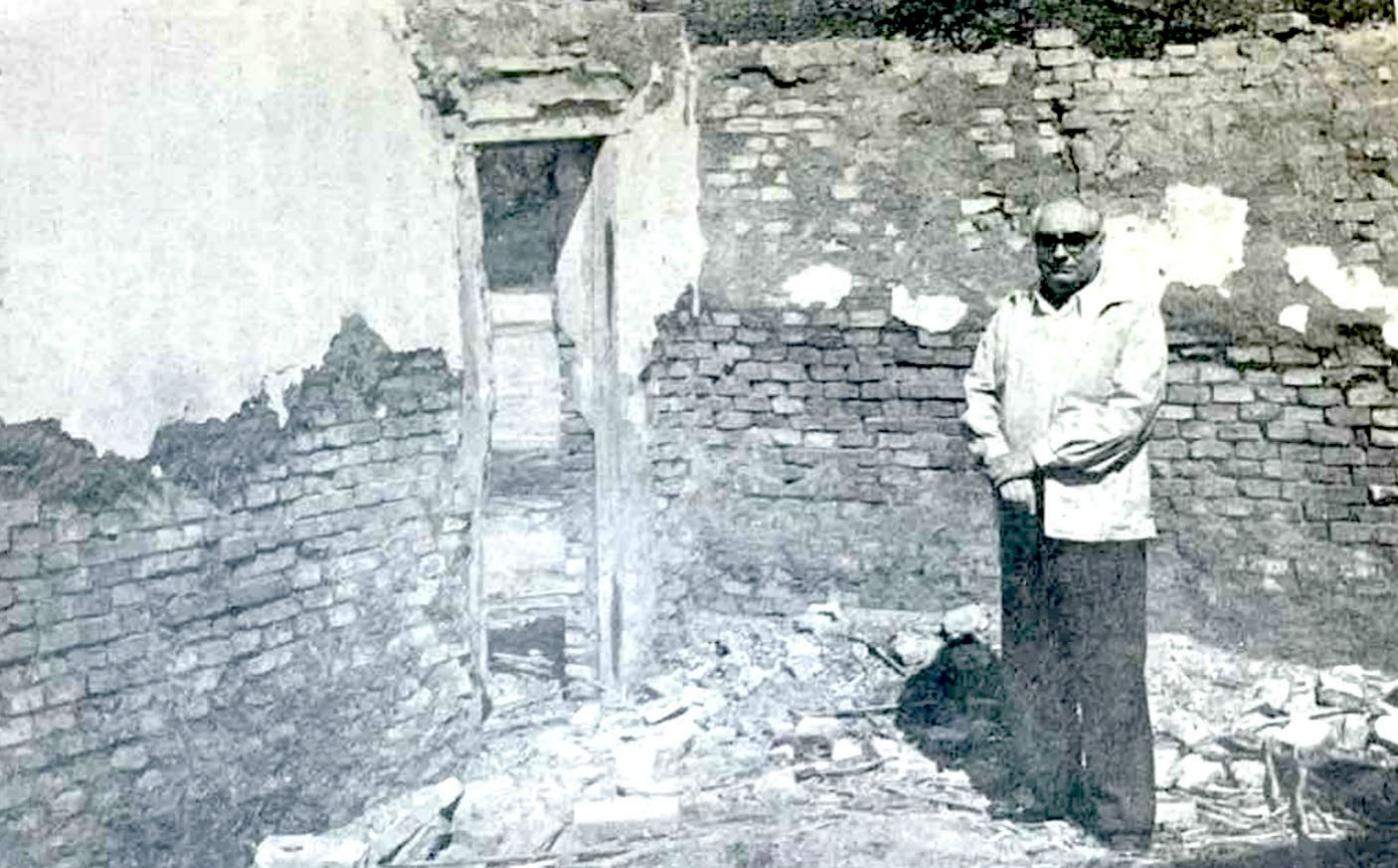
Ábalos poses for a portrait inside the ruins of the one-room schoolhouse that he once ran in Puente Negro during the years of the *Latrodectus* campaign. Source: *From the personal archive of Ábalos, reprinted in* Feliciano Huerga, *Jorge Washington Ábalos* (Editorial Universitaria de Buenos Aires, 1981), p. 53.

Thus, much of the first decade of Ábalos’ scientific career was spent roving the same transects across the Chaco that he had as the *maestro bichero,* only now he went by car, and by 1950 with an Honoris Causa Doctorate from UNT. Over the course of these iterative departures and returns, he would go on to publish some 300 scientific papers, win national awards, and travel across the hemisphere to numerous international conferences. In 1969 he would even spend a year-long stint as an Associate in Arachnology at Harvard’s Museum of Comparative Zoology as a Guggenheim fellow (Fig. 1).

In the end, the formation of Ábalos’ laboratory and field practices—indeed his career trajectory as a whole—was always entangled with the constellation of experiences, social configurations, the particular ecology and *folclore* of the Chaco that shaped his time in that place. Incorporating literary sources into an examination of his practices as a life scientist illuminates possibilities for bringing literary and cultural analysis to bear on explorations of the history of biology and practices of laboratory science. It reveals the potentially fruitful perspectives to be gained from contrapuntal approaches to a social history of field practice in geographic spaces long considered to be at (or outside) the periphery of traditionally conceived hubs of scientific knowledge production, and metrics of national “progress.” Recalling Ábalos’ years at the Universidad Nacional de Córdoba—his final post prior to his death in 1979—a colleague from the Center for Applied Zoology remembered the arrival of the *maestro* turned *científico* at his institution:

[His arrival] marked the start of an important era in the advancement of zoology [here]. Ábalos also demonstrated that it’s not always necessary to belong to the most prestigious institute, or follow the most fashionable or accepted orthodoxies of practice. One can develop their own original and creative ways of doing scientific work that are rooted in a strong connection to the needs of the region to which that work pertains. (Gardenal et al. 2018, 4:30–54)

In one light, the life’s work of Ábalos can be interpreted as a call for recognizing the existence of important moments in the history of science that came “from the periphery” and made their mark on global circuits of knowledge production. But if we can pause for a moment and take the *joven maestro/científico*’s iterative series of departures and returns as more of an interpretive framework, rather than strict delineation of movement through space over time, it can draw us to question a strict compartmentalization between that which is periphery and that which is core in this historical episode. In this vein, perhaps the story of Ábalos and his cyclical movements up and down the Dulce and Salado Rivers can serve as an invitation to re-theorize the Gran Chaco as anything but a “peripheral” space. Rather, the *monte* was integral in an interdependent web of moving people, animals, and ideas that drove important advances in life science, tropical medicine and public health research in the 20th century. Chaqueño *folclore* shaped the way that Ábalos was able to conduct his field practice, and become integrated in these social and cultural networks of global exchange that shaped the historical landscapes of public health, tropical medicine, and life sciences research in Argentina.

Stories like that of the *maestro bichero*, the no. 502 school in Tacañitas, and the spider-collecting women of the Chaco evoke the rich potential for further, and intentionally interdisciplinary, investigations into a place that Ábalos once pegged as “Argentina’s most Latin American region” (1960, p. 124). This quip was surely his nod to the overlapping dynamics of presence and absence that historians of Latin America recognize as throughlines in the immensely diverse historical-geographical range of the hemisphere. We can imagine that as he wrote that line, he was conjuring the region’s strong Indigenous presence, and commensurately severe history of dispossession, the alienation of the *monte*’s popular classes from colonial and imperial hierarchies of power, and also the dense tapestry of languages, cosmovisions and cultural expressions—its vivid *folclore* that emerged and endured over centuries. This article has presented a case study that invites us to consider, as prompted by Gordillo, “an examination into what the Gran Chaco teaches us more broadly about places as contested processes that are also part of the planet’s terrain” (Gordillo 2021, p. 277). Toward this greater project, Ábalos is an ideal protagonist, one that allows us to see the manifold ways in which the sociospatial specificity of a place like the Gran Chaco shaped people, ideas, and practices relevant to the history of, among so many things, biology. This case study has illuminated the itineraries of multiple knowledges and their circulation, from bug-collecting schoolchildren to medical professionals who relentlessly journeyed the globe in search of new ideas that would lead to a cure for one disease or another. The densely layered and interdependent nature of these mobilities shaped a young *maestro’s* field practice and professional trajectory, and more broadly it shaped the kinds of advances in regional epidemiological studies scientists such as those in the employ of the MEPRA were able to undertake.

This story set in the Gran Chaco also invites us to think about the situated nature of scientific knowledge and its relationship to local cultural and material resources, and how these themes relate to ongoing disparities in health infrastructures in rural Global South regions today. “Global inequalities in the production and appropriation of science and technology demand that we imagine networks as lumpy” reflected John Krige—a reminder that all things are not equal when it comes to benefiting from the fruits of dazzlingly transnational, transregional networks of knowledge production like that which characterized the scientific and public health research networks of this story (Krige 2019, p. 9). There is much work to be done in problematizing and nuancing the primacy of theoretical frameworks that privilege certain kinds of knowledges and certain scales of mobility as diverse actors transport those knowledges across distances long and short. In his movements through, out of, and back to *monte*, Ábalos exemplified a trajectory made possible by a willingness to take seriously these multiple ways of knowing the Gran Chaco.

## Data Availability

No datasets were generated or analysed during the current study.

## Footnotes

1 This and all following quotations from Ábalos’ works are translations by the author.

2 For an example of this trend toward regional studies, see Vetter’s work on field science in the Great Plains and Rockies during the North American railroad boom (Vetter 2019).

3 It is possible that there are additional communities where Ábalos taught, as there is consistent discrepancy across biographical and primary sources in terms of the precise geographic inventory of his teaching career.

4 “*cabecera del departamento*”.

5 See Ábalos (1937, p. 51). Doctor-writers such as Esteban Laureano Maradona and Bartolomé Bosio are two examples of contemporaries whose work Ábalos could have had the opportunity to read in his early years as a school teacher and fledgling naturalist. Writers such as these may even have served as a model for Ábalos, in that they practiced medicine, wrote literature, and were keenly observant of local landscapes, knowledges, and cultures. See, for example: Esteban Laureano Maradona. *A través de la selva* (Talleres Gráficos de la Penitenciaria Nacional de Buenos Aires, 1937); Bartolomé Bosio. *Cartas de un médico rural: médicos, medicina y enfermos* (Buenos Aires, Colección Claridad, 1936).

6 Excerpt of personal correspondence between Ábalos and Salvador Mazza reprinted and as cited in Feliciano Huerga (1981, p. 41).

7 Correspondence dated to 1938 between Mazza and Ábalos reprinted and as cited in (Feliciano Huerga 1981, p. 41).

8 Recent historiographies attest to the role of schools and teachers in amassing natural history collections – collections which Garcia and Mayoni present as rich source material for studying the history of scientific knowledge production and transmission in Argentina. See García and Mayoni (2013); Pupio (2005).

9 Social histories of specimen collecting practices have effectively blurred, in certain scenarios, the utility and distinction between professional scientists and amateur naturalists. See for example Galka (2022).

10 Excerpt from correspondence between Ábalos and Houssay reprinted in Huerga (1981).

11 Both quotations are excerpts from Jorge Washington Ábalos (1960), reprinted and as cited in Huerga (1981, p.50).

12 Within these two dossiers see the contributions of Corsi (2023) and Cházaro (2024). Relatedly, Barahona and Raj (2022) highlight the role of “non-academic networks” that have the power to define the terrain of data collection (2022).

13 For further reading on the dense professional networks that undergirded institutions such MEPRA and IMR in Argentina, see the works of medical historian Jobino Pedro Sierra e Iglesias (1990, 1993, 1996).
